# The opioid crisis: a contextual, social-ecological framework

**DOI:** 10.1186/s12961-020-00596-8

**Published:** 2020-08-06

**Authors:** Mohammad S. Jalali, Michael Botticelli, Rachael C. Hwang, Howard K. Koh, R. Kathryn McHugh

**Affiliations:** 1 Harvard Medical School, Harvard University, Boston, MA United States of America; 2 Institute for Technology Assessment, Massachusetts General Hospital, 101 Merrimac St, Suite 1010, Room 1032, Boston, MA 02114 United States of America; 3 Grayken Center for Addiction, Boston Medical Center, Boston, MA United States of America; 4 T.H. Chan School of Public Health, Harvard University, Boston, MA United States of America; 5 Harvard Kennedy School, Harvard University, Cambridge, MA United States of America; 6 Division of Alcohol and Drug Abuse, McLean Hospital, Belmont, MA United States of America

**Keywords:** opioids, opioid use disorder, social-ecological framework

## Abstract

The prevalence of opioid use and misuse has provoked a staggering number of deaths over the past two and a half decades. Much attention has focused on individual risks according to various characteristics and experiences. However, broader social and contextual domains are also essential contributors to the opioid crisis such as interpersonal relationships and the conditions of the community and society that people live in. Despite efforts to tackle the issue, the rates of opioid misuse and non-fatal and fatal overdose remain high. Many call for a broad public health approach, but articulation of what such a strategy could entail has not been fully realised. In order to improve the awareness surrounding opioid misuse, we developed a social-ecological framework that helps conceptualise the multivariable risk factors of opioid misuse and facilitates reviewing them in individual, interpersonal, communal and societal levels. Our framework illustrates the multi-layer complexity of the opioid crisis that more completely captures the crisis as a multidimensional issue requiring a broader and integrated approach to prevention and treatment.

## Background

The alarming rise in opioid misuse over the past two and a half decades has resulted in a public health crisis, characterised most prominently by a dramatic increase in drug overdose deaths. In 2017, approximately 12 million Americans misused opioids [1] and more than 47,000 people died of opioid overdose [2]. This overdose fatality rate reflects an increase of 345% between 2001 and 2016 [3], with particularly steep annual increases in overdose fatalities since 2015. The growing opioid misuse issue was recognised as a national public health emergency by the United States Department of Health and Human Services in 2017.

Over the last several years, opioid misuse gained the attention of scholars, researchers, health professionals and politicians [4]. Many have called for a broad public health approach, but the full breadth of such a strategy has not yet been articulated or realised. While various interventions have been implemented over time, they have generally been insufficient to slow the growth of non-fatal and fatal overdoses at a national level [5]. Interventions that only target a single aspect of the issue, such as restricting opioid supply, will not be sufficient to ameliorate the opioid epidemic. This is further complicated by the rapidly evolving nature of the epidemic. For example, the widespread availability of fentanyl and fentanyl analogues beginning around 2013 has resulted in a steep escalation of overdose death rates, even as other public health indicators (e.g. prescription opioid misuse) have begun to improve.

Furthermore, although the years of steeply escalating fatalities have brought newfound attention to the harms of opioid misuse, this problem is not new. Opioid use disorder (OUD) is a disabling disorder with high levels of morbidity and mortality that has devastated families and communities for decades. Although the introduction of agonist treatments in the 1970s brought critical relief to many people suffering from this illness, few people received any treatment even prior to the current crisis [6], while increasing criminalisation of drug use diverted a high proportion of this population to the criminal justice system. Thus, the inadequate public health and societal response to the harms of opioids is longstanding and new and expanded responses are sorely needed. The complexity of the crisis is represented by the multiple spheres of influence derived from individual factors, interpersonal relationships, and community and societal influences, indicating the necessity of a broader and a more integrated approach that includes prevention, treatment and overdose rescue interventions in addition to supply reduction strategies.

In this paper, we present a social-ecological framework as an important step to conceptualise the complexity of the opioid epidemic. This framework can help inform the design of impactful interventions to curb the opioid crisis. We present our framework and provide a brief overview of the literature informing its components.

### Social-ecological framework

**Fig. 1:**
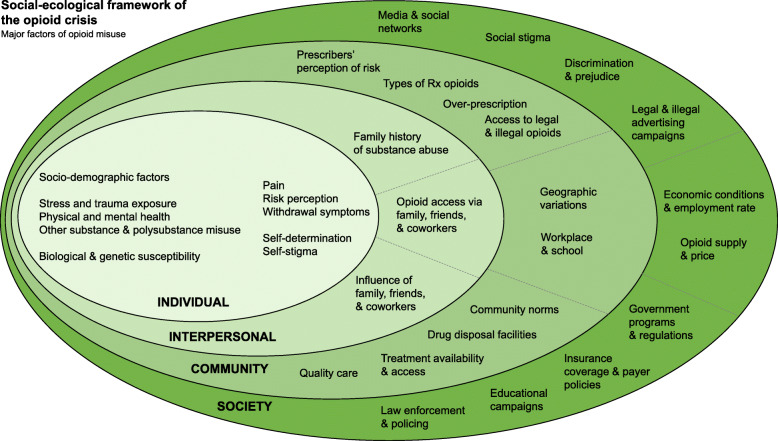
Social-ecological framework of the opioid crisis. Socio-demographic factors consist of age, race, gender, ethnicity, education, income and unemployment factors

Our social-ecological framework, illustrated in Fig. 1, depicts the major risk factors for opioid misuse on four main levels: the individual, interpersonal, communal and societal (see Additional file 1 for our use of the term ‘framework’). Each of these levels must be acknowledged to develop multifaceted and effective interventions to mitigate the opioid crisis. Following social ecological paradigms [7], prior research has presented frameworks for substance use [8] in general and alcohol use [9] in particular. While there are similarities among these frameworks and ours, there are essential factors related to opioid misuse, such as the existence of both legal (i.e. via legitimate prescription) and illegal supply sources and the availability of highly effective medications, that we discuss in this article. In the following sections, we provide a brief overview of how these levels of factors contribute to the opioid epidemic.

### Individual level

Individual-level factors in opioid misuse and OUD span sociodemographic, health and mental health, biological, and psychosocial domains. Individual factors can influence every aspect of the spectrum of opioid use and misuse, including the likelihood of exposure to opioids, initiation of opioid misuse, the development and maintenance of OUD, entry to and engagement in treatment, and relapse following an attempt to quit. These factors are complex, often interact and, in some instances, can be both a cause and consequence of opioid misuse (e.g. financial strain).

Many sociodemographic factors interact with opioid misuse, with implications for identifying at-risk populations. Opioid misuse peaks in early adulthood (approximately 18–25 years) [10]. Early initiation of opioid misuse is a significant risk factor for the development of OUD [11] and, thus, adolescence and young adulthood are key risk periods for opioid misuse. Gender can also play a role in risk for opioid misuse. For example, women are more likely than men to receive an opioid prescription [12, 13] and sex differences in the pharmacological effects of opioids have been demonstrated [14]. Critically, opioids are known teratogens and untreated OUD presents risks to both neonatal and maternal outcomes [15].

Race also plays a complex role in the opioid epidemic. People identified as non-Hispanic white are more likely to receive an opioid prescription, increasing the risk of exposure via this route [16]. Disparities in healthcare for pain often leave pain untreated or undertreated in racial and ethnic minorities [17]. Although the opioid epidemic initially predominantly affected non-Hispanic whites [18], opioid overdose is rapidly increasing among racial minorities [19]. Race also impacts access to treatment; the vast majority of studies suggest, unsurprisingly, that racial and ethnic minority groups have less access to treatment. For instance, studies show that access to effective medication for OUD is lower in communities with higher African American and Hispanic populations [20, 21]. One study found that, among people in treatment for OUD, the vast majority did not receive agonist therapies and that opioid agonist prescriptions were modestly higher in black and Hispanic clients who used heroin relative to white clients [22]. Yet, many other studies suggest that racial and ethnic minorities face disparities in access to care such as delayed admissions to treatment and lower likelihood of receiving treatment [18, 23]. Another essential component of the role of race in the opioid epidemic is the disproportionate arrest and incarceration of people of colour — we will discuss this further in the ‘Societal level’ section. Additionally, a wide array of health and mental health factors may increase the likelihood of risk for misuse, some of which overlap with those that increase the likelihood of a prescription (e.g. pain). Pain is a core element of the opioid crisis and the majority of people seeking treatment for prescription OUD report first using opioids for pain with a legitimate prescription [24]. Similarly, mental health factors are a significant contributor to opioid misuse. The majority of people with OUD also suffer from a mood or anxiety disorder [25] and psychiatric symptoms are associated with incident risk for prescription opioid misuse [26]. Additionally, a history of other substance misuse and other substance use disorders is a significant risk factor for opioid misuse; it is the most robust predictor of opioid misuse in people with chronic pain [27]. Similarly, polysubstance use increases the risk of opioid misuse [28] and recent research shows that it is highly prevalent among those with OUD [29].

A number of biological factors and genetic susceptibility can also predispose individuals to develop OUD. In addition to biological vulnerability to substance use disorders in general [30, 31], factors that influence the effects of opioids include genetic factors that alter the opioid receptors in the brain [32, 33]. Once physiological tolerance is developed to an opioid, decreases in dose or removal of the medication will result in withdrawal symptoms [34]. Although these symptoms are not fatal, they are extremely aversive and a significant reason for continued opioid use and relapse in people with OUD [35]. Indeed, over the course of OUD, the primary reason for use tends to shift to avoiding/relieving withdrawal more than managing pain or feeling good [24].

In this section, we have highlighted some key individual-level factors; however, it should be noted that the they are not meant to be comprehensive. A wide range of other psychological and temperamental factors can also play a role in the opioid epidemic; these include factors such as impulsivity [36], self-stigma [37] and self-determination [38]. Readiness for change is also another factor that is associated with entry into treatment [39] and the change process during the treatment [40], although limited data suggest this may not be related to OUD treatment outcome [41]. Overall, there is an essential need for more research on the role of these and other similar psychosocial factors.

### Interpersonal level

Family, friends and co-workers significantly shape the beliefs, attitudes and behaviours of individuals to influence the likelihood of individuals’ initiation and misuse of substances [42–44]. A family history of substance use disorder can influence opioid misuse through both genetic and environmental factors. People who have a family member with OUD are 10 times more vulnerable to misuse and overdose on the drug themselves and youth witnesses of family member overdose are more prone to overdose themselves [45, 46]. Individuals with a family history of opioid use are at a higher risk of suffering from symptoms of opioid dependence and becoming severely dependent [47]. This may be particularly important for women, for whom the risk of opioid misuse is higher when a spouse or partner misuses opioids [48]. Opioid misuse is also influenced by the accessibility to opioids from family, friends and/or co-workers. Approximately 70% of people who report non-medical opioid use reportedly obtained opioids from family members or close friends [49, 50]. Co-workers can also be a source of opioids since about 69% of people who misuse opioids are employed and 10% to 12% report drug use during working hours [51, 52].

Interpersonal relationships influence the actions of individuals to use opioids and seek treatment. Parental disapproval of drugs discourages substance use and families are often the first to detect drug misuse because of their awareness of substance history [44, 53]. Studies show that family support of recovery can increase the likelihood of receiving treatment [49, 54]. The emotional support from social supports can increase medication adherence and motivate patients during their treatment sessions [53, 55].

### Communal level

The third level of our framework considers the communal settings and their contributions to opioid-related risks [56]. The community and the immediate context in which individuals live affect their daily behaviours in significant ways. Variables such as geographic conditions, treatment accessibility, medication disposal services, workplace environment, prescribers’ perception of risk, over-prescription of opioids or under-treatment of pain, types of prescription opioid formulations available, community norms, and access to legal and illegal opioids are major risk factors that can perpetuate opioid misuse.

Between 2006 and 2017, approximately 224 million opioid prescriptions were filled annually in the United States, which is almost enough to distribute across the entire United States population [57]. Over-prescription of opioids has been influenced by several interacting factors. Oftentimes, physicians’ insufficient pain management training and knowledge on opioid misuse risk contribute to their inability to safely prescribe opioids, implement and interpret risk assessments, detect addiction, and facilitate discussions with patients [58–60]. Furthermore, prescribers who overestimate the benefits and underestimate the danger of opioids are likely to contribute to over-prescription by providing months’ worth of medication when only a few days may be needed for pain management [61, 62]. The institution of guidelines (e.g. the Centers for Disease Control and Prevention’s Guideline for Prescribing Opioids for Chronic Pain) and other interventions to improve prescribing practices has resulted in decreases in opioid prescribing [63], with reductions occurring since 2010 [57].

Over-prescription was also influenced by pharmaceutical marketing campaigns that falsely marketed opioids as non-addictive and “*create*[d] *value*” for doctors by offering monetary compensations [64]. Doctors who refused to prescribe opioids to patients were labelled as ‘opiophobic’ [65]. These incentives include sponsored meals, speaking fees, travel expenses and education [66]. Although only 7% of opioid-prescribing physicians received gifts from drug companies, they were more likely to prescribe opioids to their patients than doctors who did not benefit from the incentive [66]. Increases in prescriptions may have also reflected unintended consequences of advocacy for the improved treatment of acute and chronic pain in the 1990s, which resulted in regulatory changes requiring the assessment of pain as the ‘fifth vital sign’.

Formulations of opioids also play a role in opioid misuse. Standard opioid pills can be crushed to attain a more rapid effect via routes of administration such as intranasal or intravenous [67]. Despite the lack of sufficient supporting evidence for the efficacy of abuse-deterrent drugs in preventing misuse, the United States Food and Drug Administration has supported the development of such types of prescription opioids to address the growth in opioid-related abuse and deaths [68, 69]. The misconception that abuse-deterrent opioids are a panacea dangerously marks the issue as a pharmaceutical problem rather than a complex one integrated by biological, psychological and social challenges [67]. Furthermore, abuse-deterrent opioids do not solve the long-standing problem of heroin and other illicitly produced opioids.

The illicit market is another significant source of misused opioids. Heroin is cheap and widely available in most regions in the United States. Furthermore, there is a large online opioid market, which enables customers to purchase unregulated opioids from the web [70, 71]. The increased availability of highly potent synthetic opioids, such as fentanyl and fentanyl analogues, has contributed to the dramatic increase in rates of overdose deaths since 2015 [19].

There has been substantial geographical variation in opioid misuse and overdose, which may be attributable to a range of factors [72]. Non-metropolitan areas are known to have higher rates of opioid prescribing [73], perhaps because the rural population disproportionately consists of older adults and people employed in physically demanding jobs who may be particularly susceptible to pain-related conditions [74–77]. Overdose deaths are more prevalent in non-metropolitan areas relative to urban areas [78].

Workplaces and schools are also important settings where individuals spend significant time. Some careers have particularly high rates of opioid misuse and are typically those characterised by demanding physical labour and/or easy access to opioids; individuals involved with construction occupations suffer from the highest rate of opioid overdose [79]. Schools are also an important setting, given that adolescence is a significant risk period and diversion of medication is common in this group [80].

Community norms with respect to alcohol, tobacco and drug use can also impact the likelihood of initiation of substance misuse [72, 81]. Finally, drug disposal and collection sites can potentially deter misuse and discourage opioid diversion amongst patients’ friends and family by restricting the available supply in households and communities [44].

Similarly, the availability and access to treatment are crucial for both the adequate management of health and mental health conditions that increase risks for opioid misuse (e.g. pain, psychiatric disorders) and for the effective treatment of OUD [82]. Despite ample evidence about effective medications for the treatment of OUD [83, 84], they remain widely underutilised in the United States [85, 86] due to misperceptions about the efficacy of medications [87], policy and regulatory barriers [88], and lack of access to addiction experts [89, 90], among others. Furthermore, access to care, and to evidence-based care, varies across regions. The availability of high-quality care is also impacted by societal factors (see below). OUD is associated with high rates of relapse and the type of care received has substantial implications for outcomes [91, 92].

### Societal level

The major risk factors of opioid misuse are shaped by the larger social context, which encompass opioid supply and demand, government regulations, economic conditions and unemployment rates, elements of the media, social stigma, discrimination and prejudice, advertising campaigns, educational campaigns, and law enforcement.

The market economy of opioids is altered by the fluctuations in a drug’s supply and demand. A tremendous increase in the supply and availability of opioids arose from the over-prescription, diversion and redistribution of the pills to family, friends and/or co-workers. This was exacerbated by pharmaceutical companies’ extensive legal advertising tactics, which can lower consumers’ perception of the risks of opioids and increase their knowledge on prescription drug availability [93, 94]. Over time, the epidemic intensified as illicit opioids flooded the market and heroin became inexpensive [82, 95] — heroin is only a third of its price in the 1990s and remains cheaper than opioid prescriptions [96]. Indeed, over 80% of people who initiate heroin use first started opioid use with prescription opioids [97]; cost is one of the most commonly reported reasons for this transition [98]. Opioid supply can be managed through reduced prescribing or increased use of misuse-deterrent formulations, but these efforts can be challenged by unintended, short-term negative consequences. In particular, the decreased availability of prescription opioid analgesics can lead to increases in the use of illicitly produced opioids such as heroin [67].

Government programmes and regulations related to opioids may take many forms such as drug scheduling through the Drug Enforcement Agency, regulation of opioid prescribing practices (e.g. use of Prescription Drug Monitoring Programs; PDMPs) [99] and Medicare/Medicaid regulations. Data support the potential value of certain policies such as Good Samaritan laws [100], naloxone access legislation [101], and PDMP requirements [102]. Importantly, these different policies target different elements of the opioid crisis (e.g. overdose fatalities, prescribing practices). Government regulations also have implications for treatment availability, as federal and state governments regulate accreditation and licensing requirements as well as elements of training and service provision. For example, the Drug Addiction Treatment Act of 2000 requires that prescribers complete additional training to prescribe or dispense buprenorphine. Likewise, government regulations require that methadone is only dispensed in licensed opioid treatment programmes and cannot be used for the treatment of OUD in primary care, unlike in other countries.

The number of people who have health insurance coverage varies by state and has implications for access to OUD treatment. Medicaid expansion has played a significant role in access to medication for OUD; states that elected to expand Medicaid as part of the Affordable Care Act had a more than four-fold higher increase in prescribing of effective medications for OUD (specifically buprenorphine and naltrexone) relative to non-expansion states [103]. In addition to their contributions to the opioid supply, payer policies also impact access to treatment for pain, psychiatric illness and OUD. For example, prior authorisation for buprenorphine prescribing has been presented as a strategy for reducing diversion or other adverse events; however, this can also present a significant barrier to care [104].

Social stigma, the misconception of substance misuse as a by-product of weak willpower and moral corruption, is a significant barrier to seeking help for opioid misuse [3, 49, 105]. Likewise, cultural and social beliefs communication via media and social media can be either harmful (e.g. influencing an increase in substance use) [106, 107] or protective (e.g. increase public awareness about opioids and their potential harms).

The rise in ‘deaths of despair’ (typically referring to overdose and suicide fatalities) between 1999 and 2015 has been linked to poor economic conditions [82, 108]. During macroeconomic slumps, every percentage point increase in unemployment saw a 3.6% rise in opioid death rates and emergency visits. The fall in the employment rate resulted in lower life satisfaction and higher drug use among the population [109, 110]. A recent working paper from the National Bureau of Economic Research concluded that 10% of the rise in opioid-related deaths could be explained by recessions [111]. Nonetheless, macroeconomic impacts on drug use are complex due to the many variables affected by poor economic conditions (e.g. drug prices, incomes, employment, etc.) [112].

Law enforcement and the criminal justice systems are other significant components of the response to the opioid crisis. Law enforcement (along with other emergency responder groups) has been increasingly involved in overdose-rescue efforts. Some departments have expanded these efforts to include linkage to treatment and other supports. Law enforcement also plays a role in policing of the illicit opioid supply [113]. Finally, opioids are controlled substances that carry significant criminal penalties for possession and distribution. Substance use disorders are common among incarcerated people and release from prison is associated with a significantly heightened risk for fatal overdose [114]. Racial and ethnic minorities are disproportionately affected by the criminalisation of substance use, rather than a public health approach. Additionally, those recently released from prison were more likely to die from overdose than those who did not face the law enforcement [82, 115].

## Conclusion

The primary goal of this article was to emphasise that the opioid crisis is a multi-faceted and ever-evolving issue, which requires the consideration of numerous interacting factors in developing interventions and evaluating their effectiveness. Although much of our focus in this paper is on recent findings and trends, it is essential to note that the devastating impact of opioid misuse and OUD has been ongoing for decades. The complex and interacting contributors have evolved over time, yet many have been longstanding across each of these levels (e.g. individual, community). These factors intersect with several disparate stakeholder groups, including healthcare providers, government and regulatory agencies, insurers, and law enforcement and criminal justice, among others.

Although we have organised our framework according to the individual, interpersonal, community and society contexts, we also recognise that there is substantial interconnectedness among these contexts. For example, access to opioids — a substantial contributor of likelihood of use — cuts across each of these contexts, including the individual (e.g. presence of a pain condition), interpersonal (e.g. access to opioids from family or friends), community (e.g. availability of drug disposal resources) and society (e.g. PDMP laws). The ultimate utility of this framework is to use it to investigate the complex and multi-directional links among the factors that contribute to the ongoing epidemic.

The development of effective opioid prevention and treatment interventions requires a broad analysis of the factors that arise from multiple contexts (individual, interpersonal, community and society). We conceptualised this complex system using the social-ecological framework presented in Fig. 1. As research continues to evolve on these factors and their contribution to the opioid epidemic, this framework can be further refined. The framework is also intended to provide context for the generation of testable hypotheses about these factors, their interaction and the impact of treatment or policy levers at each level on the opioid epidemic.

## Supplementary information

**Additional file 1.**

## Data Availability

Not applicable.

## Abbreviations

OUD: Opioid use disorder
PDMP: Prescription Drug Monitoring Program
