# Supplementary material for: The opioid crisis: a contextual, social-ecological framework
Source: Health Res Policy Syst. 2020 Aug 6;18:87. doi: 10.1186/s12961-020-00596-8 (PMC7409444; doi:10.1186/s12961-020-00596-8)
Supplement: Supplementary file 1 — Additional file 1. [file 12961_2020_596_MOESM1_ESM.docx]

# ***Additional file for***

**The Opioid Crisis: A Contextual Framework and Call for Systems Science Research**

## **Our use of the term “framework”**

We presented our conceptualization of the opioid crisis and called it a ‘framework’, not a ‘model.’ It should be noted that a ‘model’ is a tool used to generate hypotheses, explain findings, and assess whether hypothesized relationships can be confirmed or disconfirmed with empirical data. Accordingly, we do not intend to call our framework a model, as a model that includes everything may be comprehensive, but it may also be unhelpful for modeling analyses. Instead, we called it a contextual framework and used it to illustrate the complexity of the crisis and stimulate discussions for future research and model-based studies.

## **Simulation modeling and analysis tools**

Several tools are utilized to incorporate into decision-making processes of policy intervention designs. These tools include but are not limited to causal loop diagrams and stock and flow diagrams. Causal loop diagrams help conceptualize the complexity and the interconnections between different elements. This qualitative method is widely used in the public health domain (e.g., see [1-3]) and incorporates positive and negative feedback loops to illustrate the different causal aspects of the problem that perpetuate, reinforce, and balance the system. Stock and flow diagrams and system archetypes are quantitative models which capture the movement and behavior of the elements within the system (e.g., see [4-7] for applications in public health and health policy). See [8] for more information about systems science tools used in public health.

## **Steps of the modeling process**

Figure A1 presents the steps of the modeling process. This process is not a linear sequence of steps and is iterative. One needs to start from step one (problem articulation) and follow the cycle but the results of any step can increase the understanding of prior steps; accordingly, any earlier step can be revised. See [9] for more information about each step and guidelines to develop a proper model.

**Iterative process:** Results of any stage can yield insights to revise any ‘*earlier’* step.

Figure A1: Modeling process (adapted from [9])

**References**:

1. Jalali, M., et al., *Dynamics of Implementation and Maintenance of Organizational Health Interventions.* International Journal of Environmental Research and Public Health, 2017. **14**(8): p. 917.

2. Jalali, M., et al., *Social influence in childhood obesity interventions: a systematic review.* obesity reviews, 2016. **17**(9): p. 820-832.

3. Jalali, M.S. and J.P. Kaiser, *Cybersecurity in Hospitals: A Systematic, Organizational Perspective.* J Med Internet Res, 2018. **20**(5): p. e10059.

4. Jalali, M.S., et al., *Dynamics of intervention adoption, implementation, and maintenance inside organizations: The case of an obesity prevention initiative.* Social Science & Medicine, 2019. **224**: p. 67-76.

5. Ghaffarzadegan, N., A. Ebrahimvandi, and M.S. Jalali, *A dynamic model of post-traumatic stress disorder for military personnel and veterans.* PloS one, 2016. **11**(10): p. e0161405.

6. Ghaffarzadegan, N., et al., *Model-Based Policy Analysis to Mitigate Post-Traumatic Stress Disorder*, in *Policy Analytics, Modelling, and Informatics: Innovative Tools for Solving Complex Social Problems*, J.R. Gil-Garcia, T.A. Pardo, and L.F. Luna-Reyes, Editors. 2018, Springer International Publishing: Cham. p. 387-406.

7. Jalali, M., et al. *Dynamics of obesity interventions inside organizations*. in *The 32nd International Conference of the System Dynamics Society*. 2014.

8. Peters, D.H., *The application of systems thinking in health: why use systems thinking?* Health Research Policy and Systems, 2014. **12**(1): p. 51.

9. Sterman, J.D., *Learning from evidence in a complex world.* Am J Public Health, 2006. **96**(3): p. 505-14.
